# Pilot Study Assessing the Baseline Physical Activity and Knowledge of Heart Health of Elementary School-Aged Children in Rural South Carolina and the Impact of an Interactive Educational Program

**DOI:** 10.7759/cureus.67802

**Published:** 2024-08-26

**Authors:** Anna C Deal, Madison Dudick, Maya Aboutanos, Nicholas Minner, Carsten Steinmetz, David Redden, Alexis M Stoner

**Affiliations:** 1 Preventive Medicine and Public Health, Edward Via College of Osteopathic Medicine, Spartanburg, USA; 2 Research and Biostatistics, Edward Via College of Osteopathic Medicine, Auburn, USA

**Keywords:** public health education, community obesity, nutrition education, awareness of cardiovascular disease, pediatric, rural area

## Abstract

Purpose: This study asked whether, if provided with education on heart-healthy habits, elementary school children in Abbeville, Greenwood, and Saluda counties in South Carolina would retain and desire to implement healthy nutrition and increased exercise. We hypothesized that teaching children about heart-healthy habits would increase their activity levels and improve their desire to be active.

Methods: This was a longitudinal survey study. Children at local after-school programs were given a pre-survey, the Kids’ Activity and Nutrition Questionnaire (KAN-Q), to assess their activity levels, diet, and knowledge of heart health. The children had a 15-minute educational period followed by an interactive program. Students completed the same survey at each of the three sessions.

Findings: A total of 44 children answered the survey questions at the beginning of each session. Out of the 10 behavioral questions, six indicated a favorable shift towards adopting healthier habits. Whole grain consumption increased to 71% from 32% (*p*<0.01). Vegetable consumption increased from 39% to 88%, and fruit consumption increased from 68% to 92% (*p*<0.01). There was a 30% decrease in the consumption of sugary drinks (*p*<0.01), and a reduction in hours watching television (*p*=0.05). All four knowledge-based questions had an increase in correct responses across the three sessions (*p*<0.01).

Conclusion: To augment behavior, a dual approach of education on nutrition and exercise leads to improved nutrition choices and exercise habits for primary prevention of cardiovascular risk factors in elementary school-aged children. These habits improve adherence to increased physical activity and better nutrition after school.

## Introduction

Children who are obese are at a higher risk of long-term health impacts, including high blood pressure, elevated cholesterol, and type II diabetes, which contribute to cardiovascular disease [1,2]. South Carolina has a higher prevalence of childhood obesity in the age group of 6-11 years (21.7%) compared to national trends (18.8%) [3]. South Carolina is an area with a high need to improve pediatric health outcomes.

Identifying evidence-based programs to address these issues is critical in building healthy lifestyle habits. Research has shown that effective treatment and engagement to counteract childhood obesity is accomplished through a combination of physical exercise and nutrition [4]. Children face two challenges when maintaining a healthy lifestyle: proper health education and continuation of said education at home [5]. Prior research assessed the impact of repetitive nutritional education on long-term retention, concluding that nutritional education influenced school children's healthy eating habits [5-7]. Exercise-focused studies, which monitored step counts and analyzed students’ activity levels during weekdays versus weekends, indicated a significant decrease in children’s physical activity on the weekends compared to physical education during school days, supporting the effectiveness of school/after-school integrated health educational programs [8-12]. When applying a multidisciplinary approach, studies utilizing anthropometric data and weekly questionnaires demonstrated the effectiveness of a combined approach in encouraging healthy lifestyles among children [10]. One study found an increase in vegetable intake but no reduction in BMI for elementary school children after a school-based program focusing solely on nutritional education [11]. Another study conducting a multidisciplinary approach found a 6.5% decrease in excess weight of children and overall improvement in dietary habits when integrating physical and nutritional education in an elementary school-based program [12]. Current literature does not analyze the long-term retention of a combined educational and nutritional program for school-aged children.

This study’s objective was to determine whether, if provided with education on heart-healthy habits, elementary school children in Abbeville, Greenwood, and Saluda counties in South Carolina would retain and desire the implementation of heart-healthy nutrition and increased exercise.

## Materials and methods

This pilot study utilized a longitudinal community-based research design by implementing a pre- and post-survey assessment. It was approved by the Institutional Review Board of Edward Via College of Osteopathic Medicine (protocol number: 1937076-4).

A convenience sampling approach was used to recruit participants. All children aged 8-11 years old who were members of an after-school program spanning three rural counties in South Carolina (Greenwood, Abbeville, and Saluda) and did not have a pre-existing medical condition that was exacerbated by exercise were included in the study (n=44). Greenwood, Abbeville, and Saluda counties are defined as rural according to the Rural-Urban Continuum Codes 45001, 45047, and 45081, respectively [13].

The study encompassed a comprehensive evaluation of participant behavior and knowledge across three distinct hour-long sessions. Each session was conducted by three second-year medical students and included education on nutrition and physical activity. Each session was designed to cover unique topics on nutrition concepts related to cardiovascular health, followed by a snack corresponding to the lesson and an activity to encourage movement (Table 1). Participant knowledge was assessed using the Kids’ Activity and Nutrition Questionnaire (KAN-Q) (see Appendices) [7]. The KAN-Q is a validated instrument designed to measure information on the students’ activity levels and diet, as well as knowledge and attitudes toward health. This survey was administered at each of the three sessions and was utilized to quantify the students’ nutrition and exercise habits.

**Table 1:** Session breakdown of educational topic covered, food or drink tried, and activity played.

	Topic	Nutrition	Activity
Session 1	Heart Health	Fruits and Vegetables	Red Light, Green Light with finding radial pulse
Session 2	Hydration	Water with Frozen Fruit	Toilet Tag
Session 3	Mindfulness	Whole Grains	Beach Ball Yoga

Due to the longitudinal nature of the study (observing outcomes on the same student multiple times), specific statistical methods were required. To correctly test for changes across sessions when the outcome was ordinal, Freidman’s test was employed. To correctly test for changes across sessions when the outcome was a Yes/No outcome, generalized estimating equations were employed. A type I error rate of 0.05 was used for all tests. All tests were conducted using SAS 9.4 (SAS Institute Inc., Cary, North Carolina, United States).

## Results

Participants

A total of 44 children participated in the study and completed all three surveys. The majority of participants identified as female (59%, n=26) with the remaining identifying as male (41%, n=18).

Behavioral changes

Out of the 10 behavioral questions posed to the participants, six indicated a favorable shift toward adopting healthier habits (Table 2). Significant increases across the three sessions were found in the consumption of whole grains (p<0.01) and the consumption of vegetables (p<0.01) (Table 2). There was a 30% decrease (n = 11) in the number of students consuming soda, sports drinks, and juices (p<0.01), as well as a reduction in the hours spent watching television after school (p=0.05) (Table 2). No significant changes were discerned in the frequency of dairy consumption, consumption of fish, eggs, or nuts, water intake, or hours devoted to the use of computers, tablets, phones, or video games.

**Table 2:** Behavior-related multiple-choice questions were compared over time between the three sessions. Data represented as % with N = 44 and significant p-value of <0.01.

		Percentage of participants (%), (N=44)			
Question		Session 1	Session 2	Session 3	p-value
Number of times white bread, noodles, rice, etc. were eaten?	None	9	24	33	
	1-2 times	60	62	63	
	3-4 times	23	13	4	
	≥5 times	9	0	0	<0.01 *
Number of times whole Grains were eaten yesterday?	None	68	38	29	
	1-2 times	32	44	45	
	3-4 times	0	18	26	
	≥5 times	0	0	0	<0.01
Number of times milk, yogurt, or cheese consumed yesterday?	None	20	22	20	
	1-2 times	66	64	67	
	3-4 times	9	13	14	
	≥5 times	5	0	0	0.9
Number of times you ate vegetables yesterday?	None	61	18	12	
	1-2 times	34	65	66	
	3-4 times	5	18	22	
	≥5 times	0	0	0	<0.01
Number of times you ate fruit yesterday?	None	32	11	8	
	1-2 times	43	60	51	
	3-4 times	16	27	39	
	≥5 times	9	2	2	<0.01
Number of times you ate fish, nuts, eggs?	None	52	58	41	
	1-2 times	41	40	58	
	3-4 times	7	2	0	
	≥5 times	0	0	0	0.33
Number of times you drank soda, sports drink, or juice?	None	16	29	47	
	1-2 times	48	53	47	
	3-4 times	27	15	4	
	≥5 times	9	2	2	<0.01
How many times did you drink water yesterday?	None	9	0	0	
	1-2 times	27	17	12	
	3-4 times	20	55	44	
	≥5 times	43	26	46	0.1
How many hours did you watch TV yesterday?	None	20	13	22	
	0-1 hour	54	49	56	
	2-3 hours	7	34	18	
	≥4 hours	18	4	4	<0.05
How many hours did you use a computer, tablet, phone or play video games?	None	20	13	22	
	0-1 hour	54	49	56	
	2-3 hours	7	34	18	
	≥4 hours	18	4	4	0.09

Knowledge-based changes

All four knowledge-based questions on the survey exhibited a significant increase in the number of correct responses across the three sessions (Table 3). The participants' ability to correctly determine the proportion of their plate that should consist of fruits and vegetables increased (p<0.01). Students also showed an increase in correctly identifying the necessity of whole grains (p<0.01) and identified 1% milk as the optimal choice (p<0.01). The final question demonstrated a 36% increase (n = 16) in children’s comprehension of the recommended 60 minutes of daily exercise (p<0.01).

**Table 3:** Knowledge-based multiple-choice questions were compared between the sessions based on the percent correct. Data is represented as % with N = 44 and p<0.01 as significant.

	Percentage correct answers (%), (N=44)			
	Session 1	Session 2	Session 3	p-value
What percent of their plate should be fruits and vegetables?	9	24	33	<0.01
Most kids need some whole grains per day	45	24	45	<0.01
1% milk is the best choice for milk	30	37	69	<0.01
Sixty minutes of exercise per day is best	29	53	65	<0.01

## Discussion

Within the constraints of the study, the results indicate that a program that includes both nutrition and exercise was effective at increasing knowledge of appropriate nutrition and exercise. The study took a unique approach by teaching the importance of nutrition and physical activity at a young age in the hope that changes in current attitudes and behavior would have beneficial effects and mitigate cardiovascular disease later in life. Key impacts of the program under study were the increased consumption of whole grains and vegetables, and the knowledge of the appropriate amount of exercise each day. A healthy diet of vegetables, whole grains, and low-fat dairy products is protective against cardiovascular disease [14]. Vegetable consumption can reduce the risk of cardiovascular disease, increase fiber and potassium intake, and provide nutrients vital to overall growth [14]. Children who are more active are shown to have stronger, healthier bones and muscles, improved cognitive function and academic performance, fewer rates of anxiety and depression, and overall improved mental health and psychological well-being [15].

The results aligned with existing literature that repetition learning is vital for children’s long-term retention of information [5,16]. The same survey was distributed at each of the three sessions, ensuring the children’s attitudes and actions could be monitored over three months. The results reflected an incremental increase in behavior as the sessions continued. Prior studies focused on either exercise or nutrition educational programs to decrease modifiable risk factors for cardiovascular disease [8-9,11]. Brusseau et al.'s study on elementary-aged children in the Southwestern United States demonstrated that children are more active on school days, advocating for increased school programming for physical education [8]. Hills et al. [9] agreed with Brusseau and associates' [8] conclusions, highlighting that the decline in physical activity has contributed to metabolic and cardiovascular problems at younger ages. Davis et al.'s study focused solely on increasing vegetable intake to decrease obesity measures and blood pressure [11]. Studies that evaluated both nutrition and exercise recorded a significant decrease in weight and an increase in healthy nutritional choices [12]. The literature, thus, affirms our study’s multidisciplinary approach within an after-school program and further correlates with our results exhibiting improvement in healthier food choices and lifestyle in elementary-aged children.

While this study found significant results, there were limitations that should be noted. The sample included a large age range of children, which made completing the survey more difficult because some children required additional assistance. Although the KAN-Q survey does include images to portray different foods and drinks, children are potentially unaware of the specific types of foods they are consuming, such as specific milk fat percentages or types of bread. Our sample size was not as robust as desired, because it was limited to the students available in the after-school program. Further, the Hawthorne effect could have been present, as the children answered the questions in the presence of the medical students who administered the survey.

Potential future research could expand upon the topics we assessed to ask additional questions about exercise, such as what type of exercise and how much exercise each day, particularly on the weekends. Future research could extend the study over the years to increase healthy habits and to measure how much information was retained. Further, in a longer-term study, it would be beneficial to include biological measurements such as heart rate, blood pressure, and waist circumference to document physical findings. These measurements could be a better way to understand how the nutrition changes and increased physical activity affected the children.

## Conclusions

The study demonstrated that an educational program organized during an after-school program positively impacted children’s ability to effectively retain and implement heart-healthy habits of balanced eating and exercise. Furthermore, the study promoted, shaped, and improved attitudes toward living a heart-healthy life.

The results demonstrated that this approach could be beneficial to after-school programs and gym classes as a template for educating elementary school-aged children. A program of this nature may be implemented in schools to combat childhood obesity in rural South Carolina as well as the nation at large.

## Appendices

**Table 4:** Kids’ Activity and Nutrition Questionnaire (KAN-Q)

Question	Answers
Yesterday, did you eat any white macaroni, noodles, bread, tortillas, or rice?	No, I did not eat any of these foods
	Yes. I ate these foods 1-2 times yesterday
	Yes. I ate these foods 3-4 times yesterday
	Yes. I ate these foods 5 or more times yesterday
Yesterday, did you eat any dark or while grain macaroni, noodles, bread, tortillas, or rice?	No, I did not eat any of these foods
	Yes. I ate these foods 1-2 times yesterday
	Yes. I ate these foods 3-4 times yesterday
	Yes. I ate these foods 5 or more times yesterday
What type of milk do you drink most of the time?	Whole milk
	2% reduced fat milk
	1% (low fat) milk
	Soy, almond, rice, or other milk
	I never drink milk
	I don’t know
Yesterday, did you eat or drink any milk, yogurt, or cheese?	No, I did not eat any of these foods
	Yes. I ate these foods 1-2 times yesterday
	Yes. I ate these foods 3-4 times yesterday
	Yes. I ate these foods 5 or more times yesterday
Did you eat any vegetables yesterday?	No, I did not eat any of these foods
	Yes. I ate these foods 1-2 times yesterday
	Yes. I ate these foods 3-4 times yesterday
	Yes. I ate these foods 5 or more times yesterday
Yesterday, did you eat any fruit?	No, I did not eat any of these foods
	Yes. I ate these foods 1-2 times yesterday
	Yes. I ate these foods 3-4 times yesterday
	Yes. I ate these foods 5 or more times yesterday
Yesterday, did you eat any fish, eggs, nuts, or peanut butter?	No, I did not eat any of these foods
	Yes. I ate these foods 1-2 times yesterday
	Yes. I ate these foods 3-4 times yesterday
	Yes. I ate these foods 5 or more times yesterday
Yesterday, did you drink any regular (not diet) sodas, sports drink, juice box, or other sugary drink?	No, I did not drink any of these
	Yes. I drank these drinks 1-2 times yesterday
	Yes. I drank these drinks 3-4 times yesterday
	Yes. I drank these drinks 5 or more times yesterday
Yesterday, did you drink any water?	No, I did not drink any water
	Yes. I drank water 1-2 times yesterday
	Yes. I drank water 3-4 times yesterday
	Yes. I drank water 5 or more times yesterday
How many hours did you watch TV when you were not in school yesterday?	I did not watch TV
	0-1 hours
	2-3 hours
	4 or more hours
How many hours did you use a computer, tablet, phone, or play video games when you were not in school yesterday?	I did use any of these devices
	0-1 hours
	2-3 hours
	4 or more hours
How much of your plate at meals should be fruits and vegetables?	None
	Some
	About half
	Most
	All
	I don’t know
How much of the grains that most kids eat should be made of whole grains?	None
	Some
	About half
	Most
	All
	I don’t know
What type of milk should most kids drink most of the time?	Whole milk
	2% reduced fat milk
	1% (low fat) or soy milk with calcium
	I don’t know
How many minutes of physical activity or exercise should most kids get each day?	15 minutes or less
	30 minutes
	45 minutes
	60 minutes (1 hour)
	I don’t know
How do you feel about eating fruit?	I really like to eat fruit
	I kind of like to eat fruit
	I don’t like to eat fruit
	I really don’t like to eat fruit
	I am not sure if I like to eat fruit
How do you feel about eating vegetables?	I really like to eat vegetables
	I kind of like to eat vegetables
	I don’t like to eat vegetables
	I really don’t like to eat vegetables
	I am not sure if I like to eat vegetables
How do you feel about eating foods made with whole grains, like brown rice or dark bread?	I really like to eat whole grain foods
	I kind of like to eat whole grain foods
	I don’t like to eat whole grain foods
	I really don’t like to eat whole grain foods
	I am not sure if I like to eat whole grain foods
How do you feel about drinking milk low in fat, like fat free or 1% milk?	I really like to drink low fat milk
	I kind of like to drink low fat milk
	I don’t like to drink low fat milk
	I really don’t like to drink low fat milk
	I am not sure if I like to drink low fat milk
How do you feel about having drinks low in sugar, like water or plain white milk?	I really like drinks low in sugar
	I kind of like drinks low in sugar
	I don’t like drinks low in sugar
	I really don’t like drinks low in sugar
	I am not sure if I like drinks low in sugar
How do you feel about doing physical activity?	I really like to do physical activity
	I kind of like to do physical activity
	I don’t like to do physical activity
	I really don’t like to do physical activity
	I am not sure if I like to do physical activity
